# CF Tummy Tracker: A Cystic Fibrosis–Specific Patient-Reported Outcome Measure for Daily Gastrointestinal Symptom Burden

**DOI:** 10.1016/j.mcpdig.2025.100203

**Published:** 2025-03-03

**Authors:** Rebecca J. Calthorpe, Hisham A. Saumtally, Laura M. Howells, Natalie J. Goodchild, Bethinn C. Evans, Zoe Elliott, Bu’Hussain Hayee, Siobhán B. Carr, Caroline M. Elston, Alexander A.R. Horsley, Daniel G. Peckham, Helen L. Barr, Giles A.D. Major, Iain D. Stewart, Kim S. Thomas, Alan R. Smyth

**Affiliations:** a School of Medicine and NIHR Nottingham Biomedical Research Centre, Nottingham, United Kingdom; b Leeds Institute of Medical Research, University of Leeds, United Kingdom; c Centre of Evidence Based Dermatology, University of Nottingham, Nottingham, United Kingdom; d Patient representative; e King’s College Hospital NHS Foundation Trust, London, United Kingdom; f National Heart and Lung Institute, Imperial College, London, United Kingdom; g Royal Brompton Hospital, part of GSTT, London, United Kingdom; h Division of Infection, Immunity and Respiratory Medicine, University of Manchester, Manchester, United Kingdom; i Nottingham University Hospitals NHS Trust, United Kingdom; j Centre Hospitalier Universitaire Vaudois, Lausanne, Switzerland; k Margaret Turner Warwick Centre for Fibrosing Lung Disease, National Heart and Lung Institute, Imperial College London, United Kingdom; l School of Medicine, Dentistry and Biomedical Sciences, Queen’s University Belfast, United Kingdom and NIHR Nottingham Biomedical Research Centre, Nottingham, United Kingdom

## Abstract

**Objective:**

To develop a cystic fibrosis (CF)–specific patient-reported outcome measure (PROM) to measure the daily burden of gastrointestinal symptoms for people with cystic fibrosis (pwCF) aged 12 years and older and address the lack of validated outcome measures for gastrointestinal symptoms in CF.

**Patients and Methods:**

CF Tummy Tracker was developed through a 5-stage approach in accordance with regulatory guidance. This included development and refinement of a conceptual framework; item generation; refinement; reduction; selection; and initial PROM testing. A mixed-methods approach, consisting of expert panel discussions, a focus group, interviews, and an online survey, was used. In initial testing, participants completed the PROM daily for 14 days via a smartphone application. This study was performed from March 14, 2022, December 12, 2023.

**Results:**

The CF community were involved throughout the development via a focus group (n=7 pwCF), interviews (n=11 pwCF), and an online survey (n=180 pwCF). A formative model was confirmed for the PROM. The final PROM, CF Tummy Tracker, consists of 10 items capturing gastrointestinal symptom burden, tested in 151 pwCF. The PROM reported no floor or ceiling effects, high test–retest reliability (intra-class correlation coefficient=0.94), and strong correlation with the anchor question.

**Conclusion:**

CF Tummy Tracker aims to address the gap in validated CF-specific PROMs for daily completion. Further testing of the psychometric properties of the PROM are planned in a new patient cohort to validate its use in clinical trials and support its use in both electronic and paper formats to increase accessibility.

Gastrointestinal symptoms including abdominal pain, bloating, and altered stool consistency are prevalent in people with cystic fibrosis (pwCF) and significantly impact on quality of life, with 2 in 3 pwCF reporting missing school or work because of these symptoms.^1^ People with CF are at risk of developing serious gastrointestinal complications including meconium ileus (affecting ∼10% of newborn infants with cystic fibrosis [CF]) and distal intestinal obstruction syndrome. Approximately 82% of pwCF experience pancreatic insufficiency, requiring pancreatic enzyme replacement therapy (PERT) with every meal.^2^ The introduction of cystic fibrosis transmembrane conductance regulator (CFTR) modulators, particularly elexacaftor/tezacaftor/ivacaftor, has improved respiratory health and life expectancy for eligible pwCF^3^; however, their gastrointestinal effects are not fully determined.

Reducing gastrointestinal symptoms and understanding extrapulmonary effects of modulators are key research priorities.^4^ However, a challenge is the lack of validated patient-reported outcome measures (PROMs) specifically development within the CF population for clinical trials. The US Food and Drug Administration^5^ and COnsensus-based Standards for the selection of health Measurement INstruments guidance^6^^,^^7^ recommend PROMs have short recall periods, and where the PROM is designed to evaluate treatment efficacy^8^ or to record symptoms such as pain, which have substantial day-to-day variability, a 24-hour recall period is preferred.^9^ However, there are relatively few CF-specific PROMs and most have recall periods of weeks, rather than days. Shorter recall periods could be advantageous in the context of early elexacaftor/tezacaftor/ivacaftor treatment where some patients report transient worsening of symptoms, which may not be adequately captured by 2-week recall.

This study aimed to develop, in conjunction with the CF community, an electronic PROM to capture daily gastrointestinal symptom burden in pwCF. This will complement existing PROMs with longer recall periods by providing insights into daily symptom variability not captured by current measures.

## Patients and Methods

### Study Design and Recruitment

**Figure 1:**
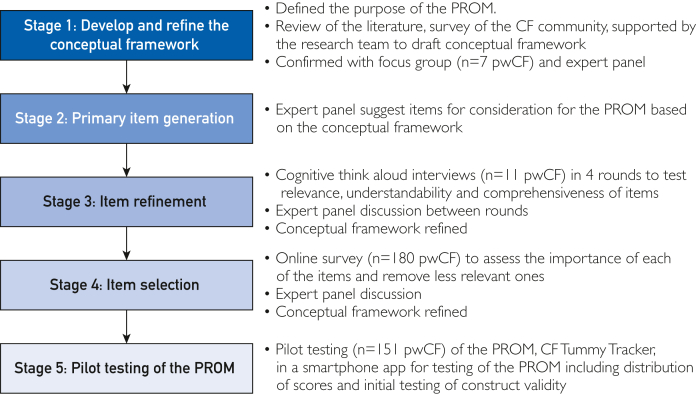
Stages of PROM development for CF Tummy Tracker. CF, cystic fibrosis; PROM, patient-reported outcome measure; pwCF, people with cystic fibrosis.

CF Tummy Tracker was developed through 5 stages (Figure 1) using a mixed-methods approach following COnsensus-based Standards for the selection of health Measurement INstruments^6^^,^^7^ and Food and Drug Administration guidance.^5^^,^^8^ An expert panel guided study development, comprising CF researchers and those with experience in PROM development methodology, CF clinicians, a gastroenterologist, and 3 members of the CF patient community. Meetings were held online to mitigate the crossinfection risks for pwCF and to broaden accessibility.

Study participation was promoted through online platforms such as social media, email, and CF organizations including CF Europe, CF Australia, and the US CF Foundation to promote international involvement, with recruitment also through participating UK CF centers. Targeted recruitment aimed for a diverse and representative sample of pwCF and participants who could engage with multiple study stages. The study received ethical approval through the UK Health Research Authority (reference: 21/NW/0345) and is registered on clinicaltrials.gov (NCT05251467).

### Stages of PROM Development

#### Stage 1—Aim: Develop the Conceptual Framework

The PROM construct of interest was “experience of gastrointestinal symptom burden,” encompassing gastrointestinal symptom severity and associated impact for pwCF. The target population was pwCF aged 12 years and older, able to answer questions independently or with minimal assistance. It was designed for use in clinical trials to assess interventions on gastrointestinal symptoms and associated burden.

The conceptual framework was developed by the expert panel, informed by a systematic review of symptoms^10^ and existing PROMs, and an international survey among the CF community.^11^ It was presented in a online focus group of pwCF. The focus group discussed the impact of gastrointestinal symptom burden and reviewed the framework to ensure all relevant aspects were captured. The focus group discussion was recorded and transcribed verbatim, and key findings were shared with the expert panel. The conceptual framework was modified and finalized before item generation (stage 2).

#### Stage 2—Aim: Item Generation

On the basis of the conceptual framework, expert panel members proposed items for the PROM. These were reviewed and included, discarded, or modified by the expert panel. Related items were consolidated into umbrella questions to avoid redundancy.

#### Stage 3—Aim: Item Refinement Through Think-Aloud Interviews

Cognitive think-aloud interviews were conducted with pwCF online, in-person, or over the phone and were recorded and transcribed verbatim. Electronic consent was obtained, including parental consent and assent from those younger than 16 years. Interviews followed a semistructured guide, using think-aloud and probing techniques to test the relevance, comprehensiveness, and comprehensibility of the items and response options.

Interviews were completed in rounds. Initial data were analyzed by R.J.C., who identified potential problems that were discussed with a subgroup of the expert panel (R.J.C, L.M.H., K.S.T, A.R.S.). Items were refined between rounds with subsequent interviews focusing on whether the changes had resolved the initial difficulties. This iterative process continued until saturation was achieved, and no significant changes were needed. Items unrelated to the construct of interest or causing confusion were removed.

#### Stage 4—Aim: Item Reduction and Selection Through an Online Survey

The importance of the items was evaluated in an online survey. People with CF rated their experience of an item over the past 24 hours (part A, 5-point Likert scale), the past year (part B: yes/no), and its perceived importance (part C: 5-point Likert scale, 1, not important at all, to 5, very important). An anchor question, “In the past 24 hours, how much have tummy symptoms bothered you?” (0, not bothered you at all; 10, very severely bothered you) was included for comparison because it closely aligned with the concept of interest, was acceptable in the think-aloud interviews, and had been used in similar PROM development research.^12^

For each participant, an impact score was calculated for each item by multiplying their experience of an item over the past year by its perceived importance. Mean impact score (0-5) for each item was calculated, with subgroup analysis by modulator status. A predefined impact score of less than 2 was used to remove any items with perceived low impact, and remaining items were assessed for collinearity. Highly correlated items (≥0.8) were consolidated, retaining those with the highest impact score.

Lasso (Least Absolute Shrinkage and Selection Operator) regression was applied to identify the most important items for predicting gastrointestinal symptom burden, but avoiding overfitting.^13^ The broad scope of the global items meant certain specific, but important items were excluded when all items were considered together in the regression analysis. Therefore, items were divided into 2 groups: those assessing specific symptoms or impact and those evaluating overall impact. Regression was then performed to select the specific items for the PROM.

#### Stage 5—Aim: Initial PROM Testing in a Smartphone Application

The final PROM, CF Tummy Tracker, was integrated into a smartphone application on the uMotif platform. Participants provided electronic consent within the application and completed the PROM every 24 hours for 14 days. Items were rated on a 5-point Likert scale and assigned numerical values (0 to 4, best to worst). Total combined scores ranged from 0 to 40, and higher scores indicated worse gastrointestinal symptom burden. Additional questionnaires were administered on days 1, 7, and 12 (available for 2 subsequent days) for initial validation and user feedback (Supplementary Material 1, available online at https://www.mcpdigitalhealth.org/). Participants received emails and push notifications reminders to encourage engagement after periods of inactivity or on targeted days.

Adequate PROM completion was defined as answering at least 9 of the 10 questions. If 1 question was missed, it was assumed to have minimal impact and scored 0. Participants missing more than 1 question were not scored and excluded from that day’s analysis. Adequate study engagement was defined as completing at least 9 of the 10 questions on 10 of the 14 days. Median completion days and daily adherence were calculated with post hoc subanalysis by gender, recruitment site (participating UK CF centers vs online), and modulator use (Mann–Whitney *U* test).

Descriptive statistics assessed total score distribution and whether floor or ceiling effects occurred (greater than 15% scoring the lowest or highest possible scores). Data were collected on a validated CF-specific PROM with 2-week recall to assess construct validity.^14^ However, permission for its use has since been revoked by the license holder. Instead, Spearman correlation coefficient assessed the correlation between total scores and the anchor question, “In the past 24 hours, how much have tummy symptoms bothered you?” on days 1, 7, and 12.

Test–retest reliability was evaluated on day 7 using the question, “How much have your tummy symptoms bothered you today compared to yesterday?” (5-point Likert scale). For participants reporting stable symptoms, intra-class correlation coefficient and Spearman correlation coefficient compared total scores over 2 consecutive days. Inter-rater reliability for individual items was assessed using percentage agreement and Gwet's AC.

Responsiveness was evaluated by examining the replies to the test–retest question (5-point Likert scale: “A lot more today than yesterday” to “A lot less today than yesterday” [Supplementary Material 2, available online at https://www.mcpdigitalhealth.org/]). Changes in total scores in each of the 5 test–retest response categories were examined using Spearman correlation coefficient and median and IQR to assess how well the PROM responds to change.

## Results

### Stage 1: Development and Refinement of the Conceptual Framework

The focus group included 7 pwCF (aged 14–59; 4 males). Six participants were on CFTR modulators and 5 on prescribed PERT. Disruptive symptoms experienced by participants included bloating; unacceptable stool frequency and consistency; and urgency. Participants highlighted the impact of gastrointestinal symptoms on sleep and social situations.

**Figure 2:**
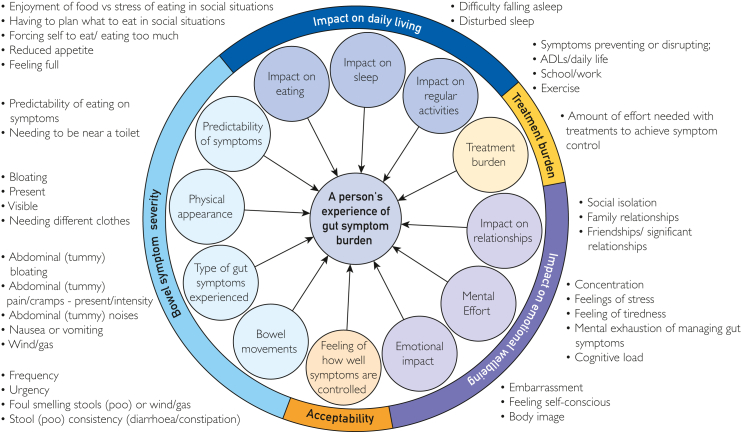
Conceptual framework of gastrointestinal symptom burden confirmed after the focus group. ADL, activity of daily living.

The conceptual framework consisted of 5 sections (Figure 2). Participants confirmed the framework was comprehensive and accurately represented gastrointestinal symptom burden but suggested refining the wording used within the overarching theme “impact on daily living” and expanding items within the “impact on eating” concept.

The group confirmed a formative model for PROM development was most appropriate as there were multiple distinctive factors that combined build a comprehensive picture on gastrointestinal symptom burden. The framework was further refined through stages 2 to 4 (Supplementary Material 3, available online at https://www.mcpdigitalhealth.org/).

### Stage 2: Item Generation

In total, 93-item ideas were suggested by the expert panel, although many were consolidated into umbrella questions. A common stem was agreed for all items: “In the past 24 hours….” Likert scales were also selected. An initial 30 questions and 2 Likert scales (numeric and descriptive) were agreed for further development (Supplementary Material 4, available online at https://www.mcpdigitalhealth.org/).

### Stage 3: Item Refinement—Think-Aloud Interviews

Think-aloud interviews with 11 participants (14-74 years; 5 males) were conducted over 4 rounds with questions refined or removed between rounds on the basis of participant feedback. Ten participants were on CFTR modulators and 10 on prescribed PERT.

Modifications included simplifying questions that combined 2 concepts. For example, “In the past 24 hours, how much has the unpredictability of tummy symptoms affected your day” was split to capture unpredictability and impact separately. Pain and discomfort were also separated because they were considered distinct concepts. Some questions were removed, particularly those that captured symptoms with minimal associated impact (eg, borborygmi) or overlapped with other concepts (eg, embarrassment).

The recall period was evaluated, and items or concepts that required a longer recall than 24 hours were removed. The recall period “24 hours” was preferred over “today” because it provided better clarity. New questions were introduced to address underrepresented concepts. Participants preferred an ordinal Likert scale, and 19 questions were finalized for stage 4.

### Stage 4: Item Reduction—Online Survey

In total, 180 participants consented to take part in the online survey, although only 17 completed the consent. Demographic characteristics are available in Supplementary Material 5 (n=163; available online at https://www.mcpdigitalhealth.org/). Not all participants responded to every question, so percentages were calculated on the basis of the number of responses for each question. Just over half (55% [n=89]) of participants were from participating UK CF centers. Adults accounted for 90% (n=146) of responses, with 9% (n=15) aged 12-15 years. Participants represented 15 countries, with the majority from the United Kingdom (79%, n=110).

### Impact Analysis

Seventeen items were considered for impact analysis, all of which scored above the predefined threshold of 2 (Table). Items were also separated by modulator status (Supplementary Material 6, available online at https://www.mcpdigitalhealth.org/).

**Table:** Impact Analysis for Each of the 17 Items for All Participants

	Frequency (mean)	Importance (mean)	All participants		
			Mean Impact score	No. of responses	Rank
Discomfort	0.88	3.78	3.52	151	1
Consistency	0.89	3.64	3.43	151	2
Pain	0.87	3.73	3.41	148	3
Bloating	0.93	3.57	3.39	144	4
Number of times open bowels	0.81	3.74	3.27	151	5
Urgency	0.84	3.49	3.12	152	6
Impact on day	0.78	3.62	3.03	145	7
Self-conscious/embarrassed	0.74	3.50	3.01	145	8
Daily routine	0.80	3.55	2.99	146	9
Unpredictability	0.85	3.39	2.91	152	10
Bloating and clothes	0.69	3.24	2.61	150	11
Sleep	0.72	3.29	2.60	148	12
Ability to cope	0.68	3.50	2.60	143	12
Concentration	0.72	3.13	2.58	143	14
Enjoyment of eating	0.66	3.55	2.53	148	15
Interactions with others	0.65	3.22	2.47	144	16
Nausea or vomiting	0.58	3.13	2.03	149	17

The items pain and discomfort, and impact on daily routine and impact on day reported high collinearity (both *r*=0.87). On the basis of their impact scores, pain and impact on daily routine were removed. Two questions on bloating were reviewed because they were felt to be similar, potentially overrepresenting the same concept. Consequently, bloating and its impact on clothes was removed owing to it combining 2 questions in 1 and a lower impact score. Two global questions impact on day and ability to cope were considered for the final PROM with impact on day chosen as this scored higher on impact analysis.

### Lasso Regression

Twelve specific items were considered for the lasso regression model (Supplementary Material 7, available online at https://www.mcpdigitalhealth.org/). The best model was identified at λ=0.073 and selected 8 variables, with a mean predicted error of 3.605.

**Figure 3:**
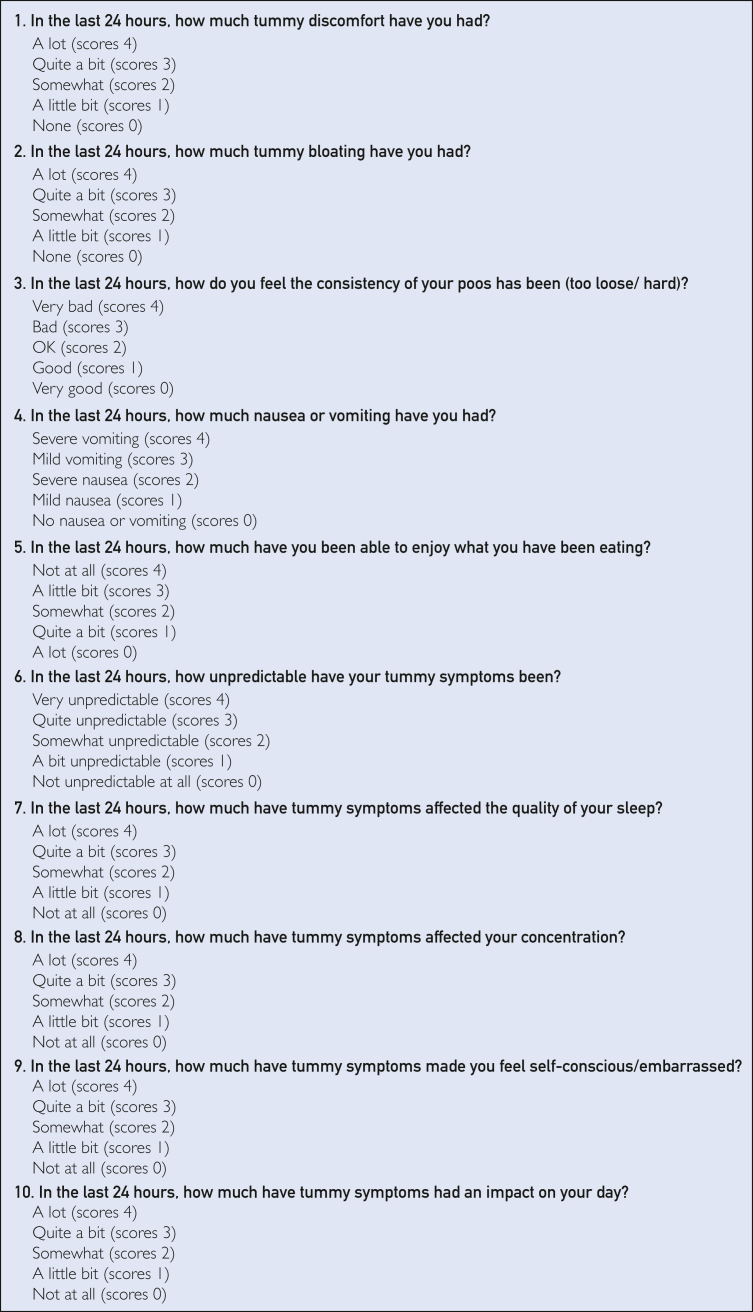
The PROM consisted of 10 questions, each contributing to the understanding of gastrointestinal symptom burden. CF Tummy Tracker is under the copyright of Nottingham University Hospitals NHS Trust.

The expert panel felt eating was not adequately represented within the final questions, despite its earlier importance. As a result, the item enjoyment of eating previously developed was added into the model. The final PROM consisted of 10 items (Figure 3), represented by the conceptual model (Supplementary Material 8, available online at https://www.mcpdigitalhealth.org/).

### Stage 5: PROM Scoring and Initial Testing

In total, 151 people consented to participate in stage 5. One was excluded owing to a fault in how their questions were displayed by the application, and only 5 completed the consent. A total of 145 participants submitted PROM data, resulting in 1378 data entry days for analysis. Demographic characteristics for those who provided PROM data were reflective of the population in which the items were developed (Supplementary Material 5). The average age was 35.8 years (range, 12-67 years), and most were identified as White (94%, 133/141). A small number (12%, n=17/141) were involved in the previous study stages. Three in 4 (76%) participants were resident in the United Kingdom.

**Figure 4:**
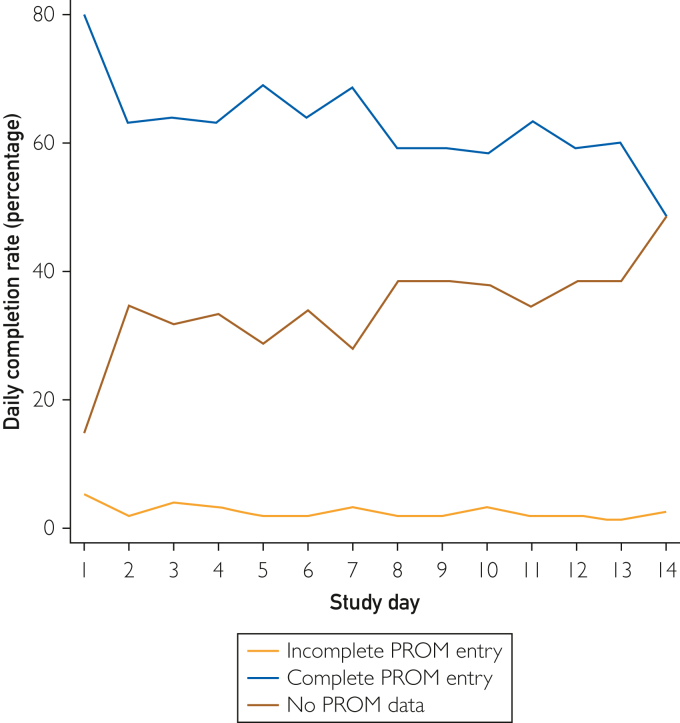
Completion rate per day within the study period. Blue, complete PROM entry; yellow, incomplete PROM entry; red, no data submited. PROM, patient-reported outcome measure.

Median daily completion was 10 of 14 days (IQR, 5-12 days), with 57% participants achieving adequate study engagement. However, engagement declined over time (Figure 4). Post hoc subanalysis revealed that daily completion was significantly higher in participants receiving care from one of the participating UK CF centers (median, 11 days; IQR, 7-13 days) compared with those recruited online (median, 10 days; IQR, 4-12 days; *P*=.018). No differences in engagement were observed on the basis of gender or modulator use.

### Daily Scores

Median total daily scores were 12 (IQR, 5-19), 8 (IQR, 3-15), and 7 (IQR, 2-13) for days 1, 7, and 12 respectively. No floor or ceiling effects were observed, although there was a rightward skew toward lower scores, indicating milder symptoms. Total scores positively correlated with the anchor question at all 3 time points (day 1: *r*=0.77; day 7: *r*=0.80; day 12: *r*=0.84; all *P*≤.001) (Supplementary Material 9, available online at https://www.mcpdigitalhealth.org/).

### Test–Retest Reliability and Responsiveness

Preliminary analysis found good test–retest reliability in participants reporting stable symptoms (n=22). Spearman correlation coefficient reported a strong positive correlation (*r*=0.90; *P*≤.001) between total scores 24 hours apart, with a mean difference of 0.136 (limits of agreement, −5.80, 6.07) (Supplementary Material 10, available online at https://www.mcpdigitalhealth.org/). Intra-class correlation coefficient using a 2-way mixed-effects model for individual absolute agreement was 0.94 (95% CI: 0.86, 0.97), indicating strong reliability. Percent agreement and Gwet's AC indicated good to very good reliability for each of the individual items (Supplementary Material 11, available online at https://www.mcpdigitalhealth.org/). Preliminary assessment of responsiveness found a moderate correlation between changes in total scores in the 5 test–retest groups (*r*=0.657) (Supplementary Material 2).

### User Feedback

User feedback was provided by 97 of the 150 participants (64%) on day 7 and 74 participants (49%) on day 12. Of those, 93% (n=90) agreed or strongly agreed that remote sign up—downloading the application, creating an account, and giving consent—was easy. Most participants agreed or strongly agreed that the questions were relevant (day 7, 90% [n=87]; day 12, 93% [n=69]), understandable (day 7, 98% [n=95]; day 12, 96% [n=71]), whereas 66% (n=64) and 73% (n=54) reported improved symptom awareness on days 7 and 12, respectively. Usability was highly rated. It was quick (day 7, 97% [n=94]; day 12, 97% [n=72]), easy to use (day 7, 97% [n=94]; day 12, 99% [n=73]), and helpful to track symptoms (day 7, 87% [n=84]; day 12, 85% [n=85]). Furthermore, 69% (n=51) indicated they would use the application again.

## Discussion

Through this research, the CF community, academics, and clinicians developed CF Tummy Tracker, a CF-specific PROM for daily recording of gastrointestinal symptoms burden. It comprises 10 questions (which can be completed quickly), being acceptable, comprehensive, and relevant to pwCF aged 12 years and older. It records daily variability, providing a snapshot of gastrointestinal symptoms. Preliminary validation found good test–retest reliability with total scores positively correlating with the construct of interest. Electronic data capture was found to be feasible. It aims to address a gap in validated CF-specific PROMs with daily recall. Although a small number of CF-specific PROMs with longer recalls exist, such as the CFAbd-Score^14^ and CF PedsQL GI,^15^ currently, the only other CF-specific PROM for daily completion is the CFAbd-day2day diary questionnaire, currently undergoing validation.^16^

We found that 57% of participants achieved satisfactory engagement. The low number of incomplete daily submissions and positive user feedback suggests the issue was potentially due to reporting fatigue rather than the application’s usability. Despite reminders sent after inactivity or on targeted days, engagement still waned throughout the study with similar rates of completion and noncompletion by day 14. A decline in electronic PROM completion over time was previously reported in a systematic review of the benefits and disadvantages of ePROMs.^17^ Our results also reflects trends in other studies involving electronic data capture in CF such as the GALAXY study, which used weekly completion of electronic PROMs to capture gastrointestinal symptomatology over a 4-week period^18^ and CFHealthHub.^19^

A strength of CF Tummy Tracker was its development through integral involvement of pwCF. A previous review of the evidence for the use of digital technology for home monitoring, adherence, and self-management in CF stressed the need for patient involvement in the design process of digital tools.^20^ Electronic recruitment methods alongside traditional ones broadened participation, whereas consent via the application enabled participants to join the study when convenient for them. However, we acknowledge that although digital technology has advantages, it also introduces the risk of digital exclusion for those without internet access or a suitable device.^21^ Future aims include making CF Tummy Tracker available in both electronic and paper formats and different languages for increased accessibility.

Limitations included the inability to assess construct validity against an existing validated PROM and a small sample size for test–retest reliability, and some participants were involved in both the development and testing stages. Further testing of the psychometric properties will be conducted in a new independent cohort alongside crosscultural validation. We acknowledge that a higher proportion of participants were females, possibly reflecting findings that report higher rates of gastrointestinal symptoms in women.^22^

## Conclusion

In conclusion, CF Tummy Tracker is a CF-specific PROM suitable for electronic use, capturing daily gastrointestinal symptom burden in pwCF. Further psychometric testing is planned to validate its use for clinical trials and additional formats to improve accessibility.

## Potential Competing Interests

Rebecca Calthorpe reports payments made to National Institute for Health and Care Research (NIHR) Programme Development Grant (PDGNIHR202952) to support salary. Alexander Horsley reports payments from NIHR Programme Development Grants (PDG) made to institution; NIHR Manchester BRC payments made to institution to support his time; Vertex Pharmaceuticals personal payment for education activities and advisory work; and NIHR Respiratory Translational Research Collaboration—chair unpaid role. Helen Barr reports grants from Cystic Fibrosis Trust and Life Arc and Cystic Fibrosis Trust and Cystic Fibrosis foundation, paid to her institution; holds PubChem Patent Summary for US-2016131648-A1 patent (Camara M, Williams P, Barrett D, Halliday N, Knox A, Smyth A, Fogarty A, Barr H, Forrester D. Alkyl quinolones as biomarkers of *Pseudomonas aeruginosa* infection and uses thereof. PubChem Internet. National Library of Medicine, National Center for Biotechnology Information; 2004; cited 2020 Nov 17; https://pubchem.ncbi.nlm.nih.gov/patent/US-2016131648-A1.); is a Member of Cystic Fibrosis Trust Research and scientific Oversight committee, NIHR translational research collaboration in Cystic Fibrosis committee, ECFS society Scientific Committee for Milan ECFS meeting 2025, and NUH bioresource access committee; and is a Chief Medical Advisor for MiDx company. Siobhán Carr reports payments from NIHR PDG made to institution; reports grants from NIHR HTA and the CF Trust. Support from Chiesi for travel and accommodation to educational meeting; and works with the CF registry and ECFS registry (no financial involvement). Alan Smyth reports payments from NIHR PDG made to institution; patent on Alkyl quinolones as biomarkers of *Pseudomonas aeruginosa* infection and uses thereof (as above); and participation on the Data Safety Monitoring Board and US Cystic Fibrosis Foundation (2019-present). Giles Major reports grants from Nestlé Health Sciences, Switzerland, and employment by Société Produits Nestlé S.A. from 2021 to 2024. Laura Howells is a co-investigator on MAGNIFY SRC funded by the Cystic Fibrosis Trust. No other conflicts of interest were disclosed by the other authors.

## Ethics Statement

The study received ethical approval through the UK Health Research Authority (reference: 21/NW/0345) and is registered on clinicaltrials.gov (NCT05251467). Electronic consent was obtained, including parental consent and assent from those younger than 16 years.
