# Supplementary material for: CF Tummy Tracker: A Cystic Fibrosis–Specific Patient-Reported Outcome Measure for Daily Gastrointestinal Symptom Burden
Source: Mayo Clin Proc Digit Health. 2025 Mar 3;3(2):100203. doi: 10.1016/j.mcpdig.2025.100203 (PMC12191005; doi:10.1016/j.mcpdig.2025.100203)
Supplement: Supplementary Materials [file mmc1.pdf]

Supplementary material

Supplementary material one: additional questionnaires used in stage 5

Anchor question (to be completed on day 1,7 and 12)
"In the last 24 hours, how much have tummy symptoms bothered you?"
11-point Likert scale from 0 (Not bothered you at all) to 10 (Very severely bothered you)

Question for test-retest reliability (day 7)
"How much have your tummy symptoms bothered you today compared to yesterday?"
5-point Likert scale:
A lot more today
A bit more today
About the same today as yesterday
A bit less today
A lot less today

Demographic information

Demographic info for app

1. How old are you? Free text
2. What is your gender? (drop down Male/Female/Prefer not to say/Self identify)
3. What is your ethnicity? (drop down with options including other and prefer not to say)
4. What country do you live in? (Drop down)
5. What is your CF centre? (free text)
6. What are your CF gene mutations? If known (drop down)
    a. DeltaF508/DeltaF508,
    b. DeltaF508/ Other mutation,
    c. Other mutation/Other mutation,
    d. Mutations unknown
7. Have you had a transplant? Multiple choice drop down list
    a. No transplant,
    b. Lung transplant,
    c. Heart transplant,
    d. Liver transplant,
    e. Kidney transplant,
    f. Other transplant)
8. Are you currently taking pancreatic enzymes, for example Creon? (Yes/No/Unsure)
9. Are you currently taking a CFTR modulator such as Kalydeco, Orkambi, Symkevi, or Kaftrio/Trikafta (Yes/No/Unsure). If yes, drop down multiple choice:
    a. Ivacaftor (Kayldeco),
    b. Lumacaftor/ Ivacaftor (Orkambi),
    c. Tezacaftor/ Ivacaftor (Symdeko/ Symkevi)
    d. Elexacaftor/ Tezacaftor/ Ivacaftor (ETI) (Kaftrio/ Trikafta)
10. Have you ever had gut surgery, including as a baby (for example for meconium ileus) (Yes/No/ Unsure)
11. Have you ever been told by your CF team that you have ever had any of the following conditions? Please select all that apply. Options:
    a. None,
    b. Meconium ileus,
    c. CF related diabetes,
    d. CF related liver disease,
    e. Distal intestinal obstruction syndrome,
    f. Pancreatitis,
    g. Unsure)
12. Have you been involved in any of the earlier development stages for CF Tummy Tracker®? (such as a focus group, interviews, online survey) (Yes/No/ Unsure)

Satisfaction questionnaires
Day 7

1. The information given to me before the trial contained everything I wanted to know (for example time commitment, what you needed to do, who to contact with questions)
    - Strongly disagree
    - Disagree
    - Neither agree or disagree
    - Agree
    - Strongly Agree

2. I found it easy to sign up to the trial (for example downloading the app, creating an account, giving consent)
    - Strongly disagree
    - Disagree
    - Neither agree or disagree
    - Agree
    - Strongly Agree

3. The questions included on the app are relevant to me
    - Strongly disagree
    - Disagree
    - Neither agree or disagree
    - Agree
    - Strongly Agree

4. The questions included on the app are easy for me to understand
    - Strongly disagree
    - Disagree
    - Neither agree or disagree
    - Agree
    - Strongly Agree

5. Using the app is helpful to be able to track my tummy symptoms
    - Strongly disagree
    - Disagree
    - Neither agree or disagree
    - Agree
    - Strongly Agree

6. Using the app has improved my overall awareness of my tummy symptoms
    - Strongly disagree
    - Disagree
    - Neither agree or disagree
    - Agree
    - Strongly Agree

7. I find the app easy to use
    - Strongly disagree
    - Disagree
    - Neither agree or disagree
    - Agree
    - Strongly Agree

8. The time taken to complete the app each day is acceptable for me.
    - Strongly disagree
    - Disagree
    - Neither agree or disagree
    - Agree
    - Strongly Agree

9. Does the app have any glitches or anything you would change?
   No
   Yes – please tell us more about this (free text)

10. Overall, how would you rate your experience of using the app?
   Very Poor
   Poor
   Ok
   Good
   Very Good

Day 14

1. The questions included on the app are relevant to me
   Strongly disagree
   Disagree
   Neither agree or disagree
   Agree
   Strongly Agree

2. The questions included on the app are easy for me to understand
   Strongly disagree
   Disagree
   Neither agree or disagree
   Agree
   Strongly Agree

3. Using the app is helpful to be able to track my tummy symptoms
   Strongly disagree
   Disagree
   Neither agree or disagree
   Agree
   Strongly Agree

4. Using the app has improved my overall awareness of my tummy symptoms
   Strongly disagree
   Disagree
   Neither agree or disagree
   Agree
   Strongly Agree
5. I find the app easy to use
   Strongly disagree
   Disagree
   Neither agree or disagree
   Agree
   Strongly Agree

6. The time taken to complete the app each day is acceptable for me.
   Strongly disagree
   Disagree
   Neither agree or disagree
   Agree
   Strongly Agree

7. Does the app have any glitches or anything you would change?
   No
   Yes – please tell us more about this (free text)

8. Overall, how would you rate your experience of using the app?

Very Poor
Poor
Ok
Good
Very Good

9. I found it easy to be able to contact the study team during the trial
    Strongly disagree
    Disagree
    Neither agree or disagree
    Agree
    Strongly Agree
    Not applicable

10. I would use this app to track my tummy symptoms in the future
    Strongly disagree
    Disagree
    Neither agree or disagree
    Agree
    Strongly Agree

11. Please tell us how you found using the app and any other feedback you have (free text)

Supplementary material two

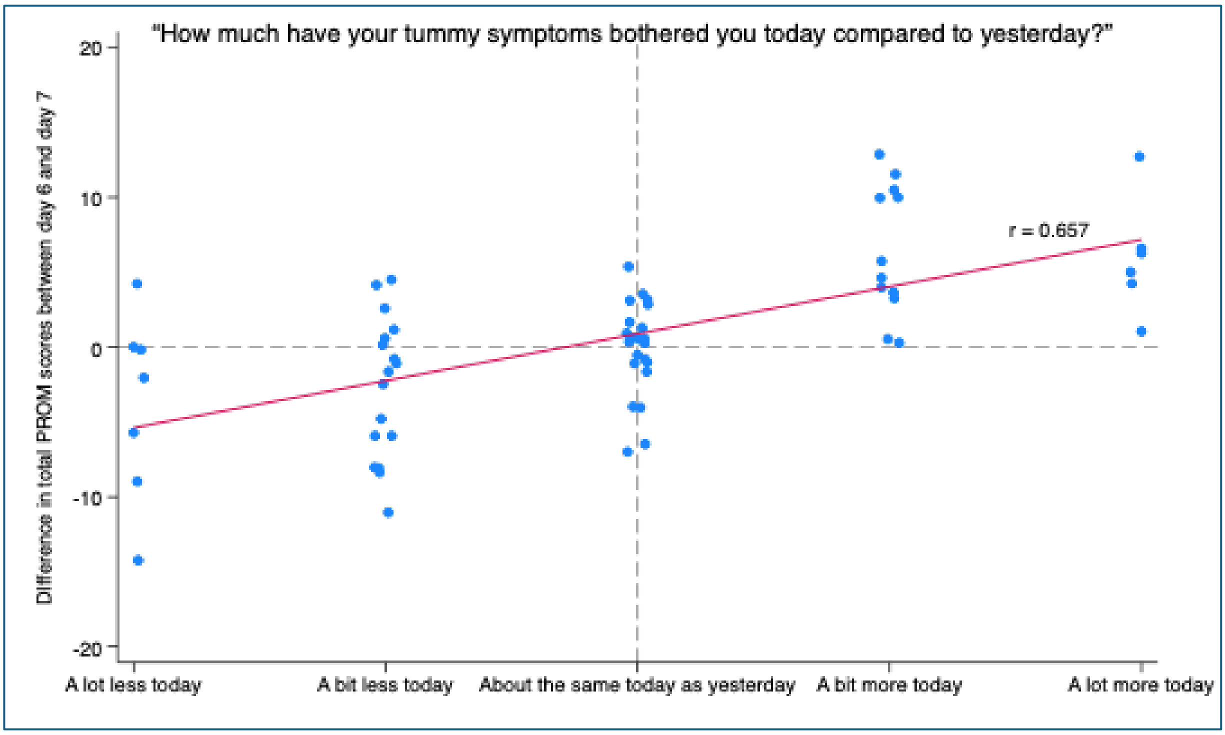

**Response options**	**Median difference in scores between day 6 and day 7**	**Mean difference in scores between day 6 and day 7**
A lot more today than yesterday (n = 6)	5.5 (IQR 4, 7)	5.83 (SD 3.66)
A bit more today than yesterday (n = 12)	5.5 (IQR 3, 10)	6.42 (SD 4.42)
About the same today as yesterday (n=22)	0 (IQR -1, 2)	-0.14 (SD 3.03)
A bit less today than yesterday (n = 17)	-2 (IQR -6, 0)	-2.76 (SD 4.52)
A lot less today than yesterday (n = 7)	-2 (IQR -9, 0)	-3.86 (SD 6.18)

Supplementary material 2. Assessment of responsiveness. Participants were asked the question "How much have your tummy symptoms bothered you today compared to yesterday?". Top: Scatter plot and spearman's correlation coeffect showing difference in total PROM scores over two consecutive days in each of the 5 categories. Bottom: Median and mean difference in total PROM scores for each of the five categories.

Supplementary material three

Change to the conceptual framework	Rationale
**Focus group**	
Amendment of concept: “impact of daily life” changed to “impact on regular activities”	Participants expressed difficulties in the distinction between the concept "impact on daily life" and the broader overarching theme "impact on daily living”. They highlighted that whilst the other concepts "laddered up" and exemplified the overarching theme, "impact on daily life" might encompass various other types of impact also captured by other concepts, potentially serving as a catch-all category. Participants felt “impact on regular activities” encompassed this concept better.
Items added: Impact on eating	Participants expressed the importance of this concept and that it needed to include both reduced and increased appetite, reflecting that pwCF experience increased appetite on starting modulator therapy. The social impact of eating with gastrointestinal symptoms and predictability of symptoms were raised as important.
Item removed: Self-esteem removed from the emotional impact concept.	Incorrect recall period. Self-esteem was felt not show daily variability and was also likely multifactorial, not limited to the impact of gastrointestinal symptoms.
**Think-aloud Interviews**	
Concept wording changed: “impact on relationships” changed to “impact on interactions”	Interviews revealed issues of the word “relationship” as it was felt not to be relevant to all and was changed to “impact on interactions” with the item “interaction with others” added to be more inclusive to those not in a romantic relationship.
Rewording of concept “feeling of how well symptoms are controlled” to “feeling of how good/ bad symptoms are”	Interviews highlighted that acceptability of symptoms was more closely aligned with how good or bad symptoms were, rather than control.
Concept removed: “treatment burden” removed	Participants felt that a recall period of 24 hours was too short for this concept and was removed.
Items removed: mental exhaustion, cognitive load, body image	Participants felt the recall period of 24 hours was too short for these items and items were removed.
Items removed: tummy noises, wind/gas and foul-smelling stools	Interviews highlighted these items either had little associated impact or impact was closely related to another concept i.e. emotional impact (embarrassment) and items were removed.
Survey	
Concepts removed: “Interactions with others”	Removed by lasso regression. Expert panel deemed this to acceptable given the degree of collinearity to other items included in the model such as impact on day (0.7) and concentration (0.79)
Concept removed: “impact on regular activities” removed during survey analysis	Item representing the concept “impact on regular activity” removed following impact analysis as high collinearity with the item “impact on day” but scored lower on the impact analysis.
Rewording of concept: “feeling of how good/ bad symptoms are” changed to “Overall impact of gut symptoms”	Modified following survey analysis to reflect a more global view of the impact on gastrointestinal symptoms, which is different for different people but may incorporate how good/ bad symptoms are, its impact on routine or interactions with others.
Concept “type of gut symptom experienced” represented by two items “discomfort” and “nausea and vomiting”	Lasso regression ruled both items contributed to the model of understanding gastrointestinal symptom burden. Expert panel were in agreement and felt both items were of clinical importance and offered something different to the model.

Item added: Enjoyment of eating	Lasso regression did not include the item “enjoyment of eating”. Expert panel discussion felt it to be an important concept to be included as it had been raised previously by participants as important had low collinearity with other included items.

Supplementary material three. Changes made to the conceptual framework during CF Tummy Tracker® development process

Supplementary material four
CARDS-CF interview questions: round one

In the last 24 hours;

Overall tummy symptoms

1. How have your tummy symptoms been?
   a. Very bad
      Bad
      Ok
      Good
      Very good

   b. 0 (Very bad symptoms) 1 2 3 4 (no symptoms at all)

   c. If 0 is very bad tummy symptoms and 4 is no tummy symptoms at all in the last 24 hours;
      0 1 2 3 4

2. How much tummy pain or discomfort have you had?
   a. Very severe tummy pain
      Severe tummy pain
      Moderate tummy pain
      Mild tummy pain
      No tummy pain

   b. 0 (very severe pain) 1 2 3 4 (no pain/discomfort)

   c. If 0 is very severe pain or discomfort and 4 is no pain or discomfort at all in the last 24 hours;
      0 1 2 3 4

3. How much tummy bloating have you had?
   a. Very bad tummy bloating
   b. Bad tummy bloating
   c. Moderate tummy bloating
   d. Mild tummy bloating
   e. No tummy bloating

   b. 0 (very bad bloating) 1 2 3 4 (no bloating)

   c. If 0 is very bad bloating and 4 is no bloating at all in the last 24 hours;
      0 1 2 3 4

Predictability of symptoms:

4. How much has the unpredictability of your tummy symptoms affected your day?
   a. a lot
      quite a bit
      somewhat
      a little bit
      not at all

   b. 0 (a lot) 1 2 3 4 (not at all)

5. To what extent has your need to poo urgently affected your day?
   a. a lot
      quite a bit
      somewhat

a little bit
not at all

b. 0 (a lot) 1 2 3 4 (not at all)

Physical appearance:

6. How much has your bloating impacted on what you were able to wear?
   a. a lot
      quite a bit
      somewhat
      a little bit
      not at all

   b. 0 (a lot) 1 2 3 4 (not at all)

7. How much has having a bloated tummy affected your day?
   a. a lot
      quite a bit
      somewhat
      a little bit
      not at all

   b. 0 (a lot) 1 2 3 4 (not at all)

Bowel movements:

8. How much has the consistency of your poos (too loose/ hard) bothered you?
   a. a lot
      quite a bit
      somewhat
      a little bit
      not at all

   b. 0 (a lot) 1 2 3 4 (not at all)

9. How much has having hard poos bothered you?
   a. a lot
      quite a bit
      somewhat
      a little bit
      not at all

   b. 0 (a lot) 1 2 3 4 (not at all)

10. How much has having loose poos bothered you?
   a. a lot
      quite a bit
      somewhat
      a little bit
      not at all

   b. 0 (a lot) 1 2 3 4 (not at all)

Type of gut symptoms experienced:

11. How much has tummy discomfort or tummy pain affected your day?

a. a lot
   quite a bit
   somewhat
   a little bit
   not at all

b. 0 (a lot) 1 2 3 4 (not at all)

12. How much has passing gas/wind affected your day?
   a. a lot
      quite a bit
      somewhat
      a little bit
      not at all

   b. 0 (a lot) 1 2 3 4 (not at all)

13. How much has abdominal noises or gurgling affected your day?
   a. a lot
      quite a bit
      somewhat
      a little bit
      not at all

   b. 0 (a lot) 1 2 3 4 (not at all)

<u>Impact on eating:</u>

14. How much has nausea or acid reflux affected your day?
   a. a lot
      quite a bit
      somewhat
      a little bit
      not at all

   b. 0 (a lot) 1 2 3 4 (not at all)

15. How much has your appetite affected your wellbeing?
   a. a lot
      quite a bit
      somewhat
      a little bit
      not at all

   b. 0 (a lot) 1 2 3 4 (not at all)

16. How much have you been able to enjoy what you have been eating?
   a. Not at all
      a little bit
      somewhat
      quite a bit
      a lot

   b. 0 (Not at all) 1 2 3 4 (a lot)

Impact on sleep:

17. How much has your tummy symptoms affected the quality of your sleep?
    a. a lot
       quite a bit
       somewhat
       a little bit
       not at all

    b. 0 (a lot) 1 2 3 4 (not at all)

18. How much have your tummy symptoms affected your sleep?
    a. a lot
       quite a bit
       somewhat
       a little bit
       not at all

    b. 0 (a lot) 1 2 3 4 (not at all)

Impact on regular activities:

19. How much have your tummy symptoms affected your daily routine?
    a. a lot
       quite a bit
       somewhat
       a little bit
       not at all

    b. 0 (a lot) 1 2 3 4 (not at all)

20. How much have your tummy symptoms influenced your activities?
    a. a lot
       quite a bit
       somewhat
       a little bit
       not at all

    b. 0 (a lot) 1 2 3 4 (not at all)

Treatment burden:

21. How much effort have you had to put in to managing your tummy symptoms?
    a. a lot
       quite a bit
       somewhat
       a little bit
       not at all

    b. 0 (a lot) 1 2 3 4 (not at all)

Impact on relationships:

22. How much have your tummy symptoms affected your relationships with others?
    a. a lot
       quite a bit
       somewhat
       a little bit
       not at all

b. 0 (a lot) 1 2 3 4 (not at all)

23. How much have your tummy symptoms affected you spending time with others?
    a. a lot
       quite a bit
       somewhat
       a little bit
       not at all

    b. 0 (a lot) 1 2 3 4 (not at all)

<u>Mental effort:</u>

24. How much have your tummy symptoms made you feel mentally tired?
    a. a lot
       quite a bit
       somewhat
       a little bit
       not at all

    b. 0 (a lot) 1 2 3 4 (not at all)

25. How much has your tummy symptoms affected your concentration?
    a. a lot
       quite a bit
       somewhat
       a little bit
       not at all

    b. 0 (a lot) 1 2 3 4 (not at all)

<u>Emotional impact:</u>

26. How much have your tummy symptoms made you feel self - conscious/embarrassed?
    a. a lot
       quite a bit
       somewhat
       a little bit
       not at all

    b. 0 (a lot) 1 2 3 4 (not at all)

27. How much have your tummy symptoms bothered you?
    a. a lot
       quite a bit
       somewhat
       a little bit
       not at all

    b. 0 (a lot) 1 2 3 4 (not at all)

28. How much have your tummy symptoms affected your emotional wellbeing?
    a. a lot
       quite a bit

somewhat
a little bit
not at all

b. 0 (a lot) 1 2 3 4 (not at all)

Acceptability:

29. How well controlled have your tummy symptoms been?
    a. not at all controlled
       a little bit controlled
       somewhat controlled
       quite well controlled
       very well controlled

    b. 0 (not at all controlled) 1 2 3 4 (very well controlled)

30. How satisfied are you with how well your tummy symptoms are controlled?
    a. not at all satisfied
       a little bit satisfied
       somewhat satisfied
       quite satisfied
       very satisfied

    b. 0 (not at all satisfied) 1 2 3 4 (very satisfied)

Supplementary material five: demographic data for stages four and five

Demographic	Percentage (online survey)	Percentage (app use)
**Age**	**(n=163)**	**(n=141)**
12 - 15 years	9% (n=15)	4% (n=5)
16-20 years	9% (n=15)	6% (n=8)
21-30 years	28% (n=45)	26% (n=37)
31-40 years	27% (n=44)	30% (n=42)
41-50 years	15% (n=25)	22% (n=31)
51-60 years	7% (n=12)	11% (n=15)
61 years and above	3% (n=5)	2% (n=<5)
Prefer not to say	1% (n=<5)	-
**Gender**	**(n=163)**	**(n=141)**
Female	64% (n=104)	74% (n=104)
Male	34% (n=55)	26% (n=37)
Prefer not to say or self-identify	2% (n=<5)	0%
**Country**	**(n=159)**	**(n=141)**
UK	79% (n=125)	76% (n=107)
Europe	13% (n=20)	9% (n=12)
Australia and New Zealand	5% (n=8)	9% (n=13)
US and Canada	3% (n=5)	6% (n=9)
Africa	<1% (n=<5)	0%
**Genotype (self-report)**		**(n=141)**
**F508 heterozygous**	-	47% (n=66)
**F508 homozygous**	-	33% (n=46)
**Other mutations**	-	8% (n=11)
**Unknown**	-	13% (n=18)
**Current PERT use**	**(n=163)**<br>87% (n=141)	**(n=141)**<br>88% (n=124)
**Current Modulator use**	**(n=163)**<br>Yes: 82% (n=133)<br>- Ivacaftor: 32% (n=43/133)<br>- Lumacaftor/ ivacaftor 1% (n=<5/133)<br>- Tezacaftor/ Ivacaftor 3% (n=<5/133)<br>- Elexacaftor/ Tezacaftor/ Ivacaftor 94% (n=125/133)<br>- Missing data 1% (n=<5/133)	**(n=141)**<br>Yes: 84% (n=118)<br>- Ivacaftor 32% (n=38/118)<br>- Lumacaftor/ ivacaftor 2% (n=<5/118)<br>- Tezacaftor/ Ivacaftor: <1% (n=<5/118)<br>- Elexacaftor/ Tezacaftor/ Ivacaftor (92% n=108/118)

**Organ transplant recipient**	**(n=161)**<br>Yes 7% (n=11)	**(n=141)**<br>Yes 8% (n=11)
**Self-reported CF-related gastrointestinal comorbidities and complications**	**(n=162)**	
Meconium ileus	15% (n=25)	16% (n=22)
Pancreatic insufficiency	69% (n=111)	-
DIOS	23% (n=38)	22% (n=31)
CF related diabetes	38% (n=61)	50% (n=70)
CF related liver disease	23% (n=37)	18% (n=26)
Pancreatitis	-	16% (n=22)
Other	6% (n=9)	-
None of the above	15% (n=24)	-

Supplementary material five. Demographic data of participants completing the online survey for item selection (stage 4) and initial testing within the app (stage 5).

Supplementary material six: Impact analysis by modulator status

	All participants			Modulator group			Non-modulator group		
	Impact score	Number responses	Rank	Impact score	Number responses	Rank	Impact score	Number responses	Rank
**Discomfort**	3.52	151	1	3.48	123	1	3.68	28	3
**Consistency**	3.43	151	2	3.46	123	2	3.29	28	5
**Pain**	3.41	148	3	3.31	121	4	3.81	27	1
**Bloating**	3.39	144	4	3.33	119	3	3.68	25	2
**Number times open bowels**	3.27	151	5	3.25	123	5	3.36	28	4
**Urgency**	3.12	152	6	3.14	125	6	3.04	27	9
**Impact on day**	3.03	145	7	3.00	120	8	3.16	25	6
**Self-conscious/ embarrassed**	3.01	145	8	3.03	119	7	2.92	26	10
**Daily routine**	2.99	146	9	2.98	120	9	3.08	26	7
**Unpredictability**	2.91	152	10	2.88	125	10	3.07	27	8
**Bloating plus clothes**	2.61	150	11	2.55	122	14	2.89	28	11
**Sleep**	2.60	148	12	2.58	121	12	2.70	27	12
**Ability to cope**	2.60	143	12	2.64	118	11	2.44	25	16
**Concentration**	2.58	143	14	2.56	119	13	2.68	25	13
**Enjoyment**	2.53	148	15	2.53	121	15	2.52	27	15
**Interactions with others**	2.47	144	16	2.45	119	16	2.56	25	14
**Nausea or vomiting**	2.03	149	17	1.97	122	17	2.33	27	17

Supplementary material six.. Impact analysis based on whole group analysis and separated by those self-reporting as taking a CFTR modulator vs those who were not.

Supplementary material seven: Lasso regression results

Lasso regression for specific questions

ID	Regularisation Parameter (λ)	Number of non-zero coefficients	Cross-validated mean predicted error	Variables add to the model
2	1.748	2	8.197	Concentration, self-conscious/ embarrassed
3	1.593	4	7.578	Consistency, discomfort
7	1.098	5	5.595	Unpredictable
9	0.912	6	5.034	Bloating
12	0.690	7	4.443	Sleep
28	0.156	8	3.625	Nausea and vomiting
36*	0.073	8	3.605	

Supplementary material seven. Lasso regression results for specific questions on symptoms or impact.
** Denotes optimal model for the regression.*

Supplementary material eight: final conceptual model for CF Tummy Tracker®

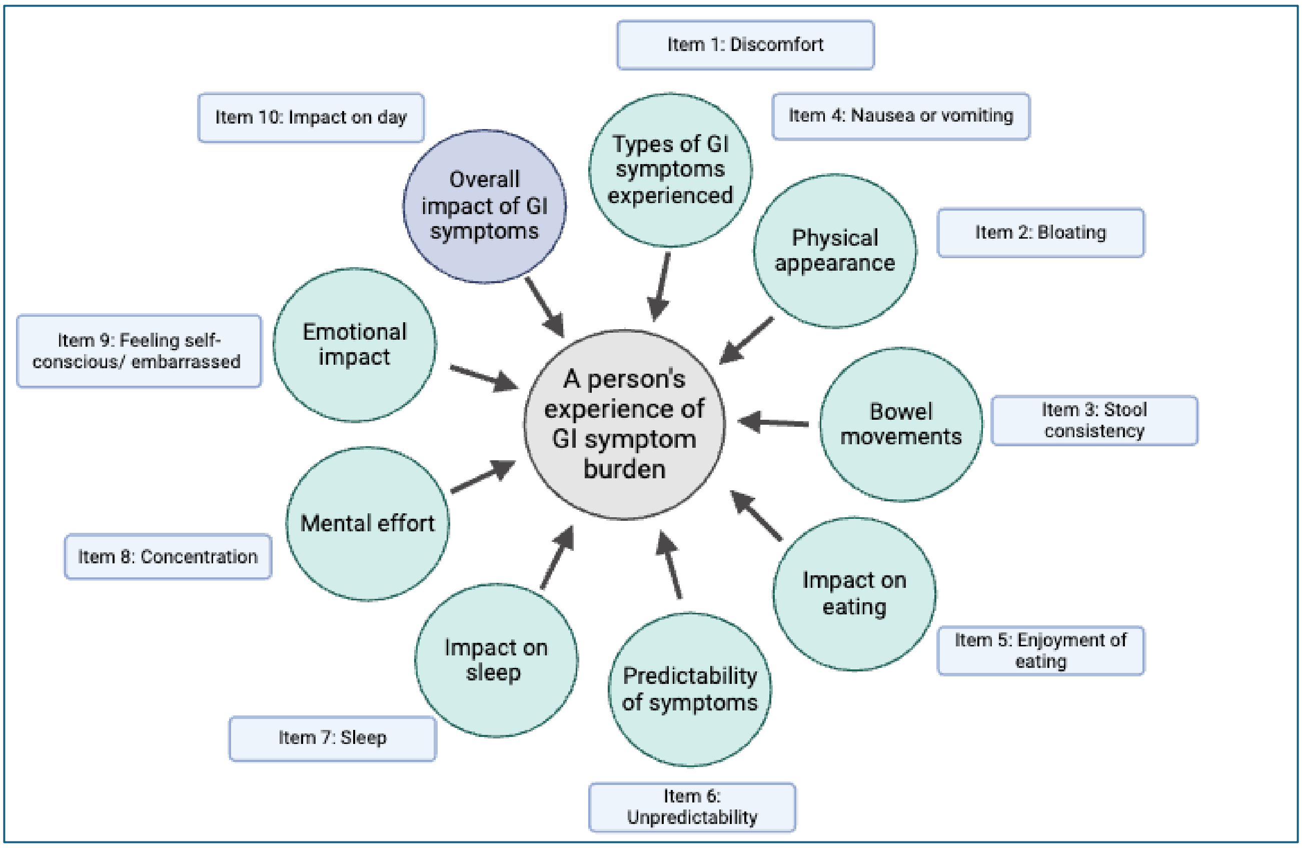

Supplementary material eight. Final conceptual framework for CF Tummy Tracker®

Supplementary material nine

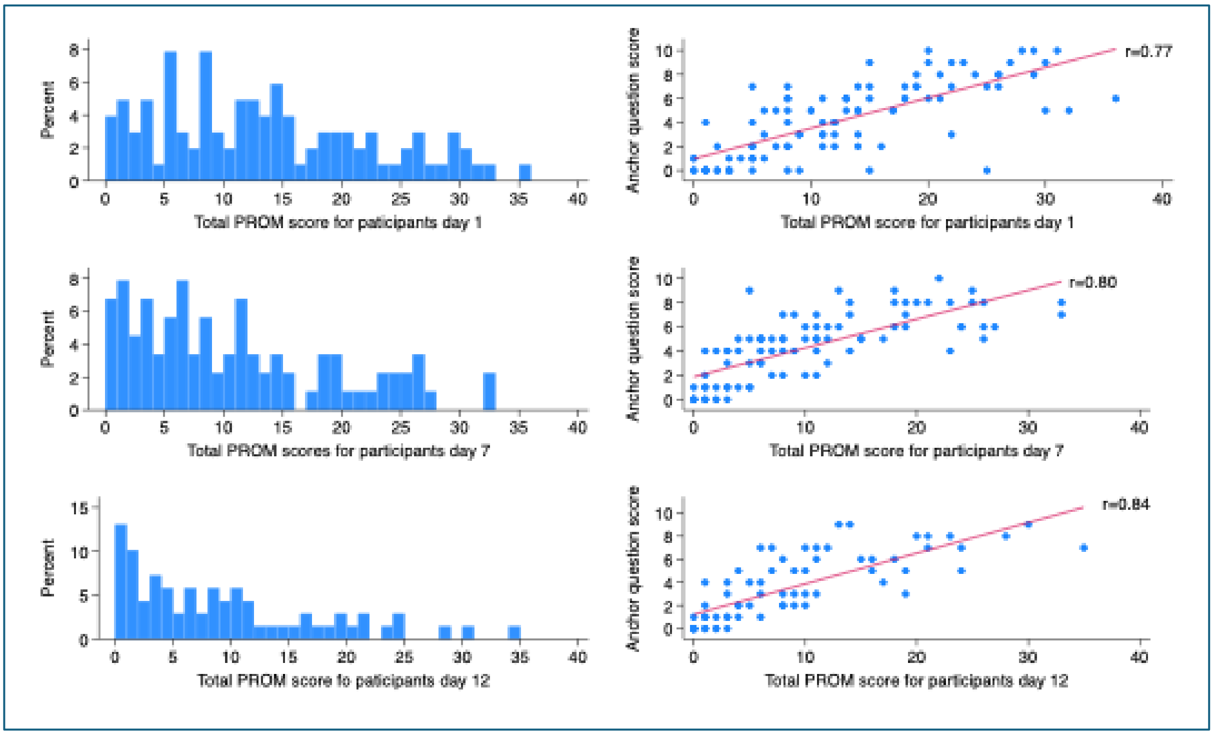

Supplementary material nine. Left (9a): Histograms showing total PROM scores for participants on day one (top row), day seven (middle row), and day 12 (bottom row) of the study period. Right (9b) Total PROM scores positively correlated with the anchor question "In the last 24 hours, how much have tummy symptoms bothered you?" at all three time points across the study period.

Supplementary material ten

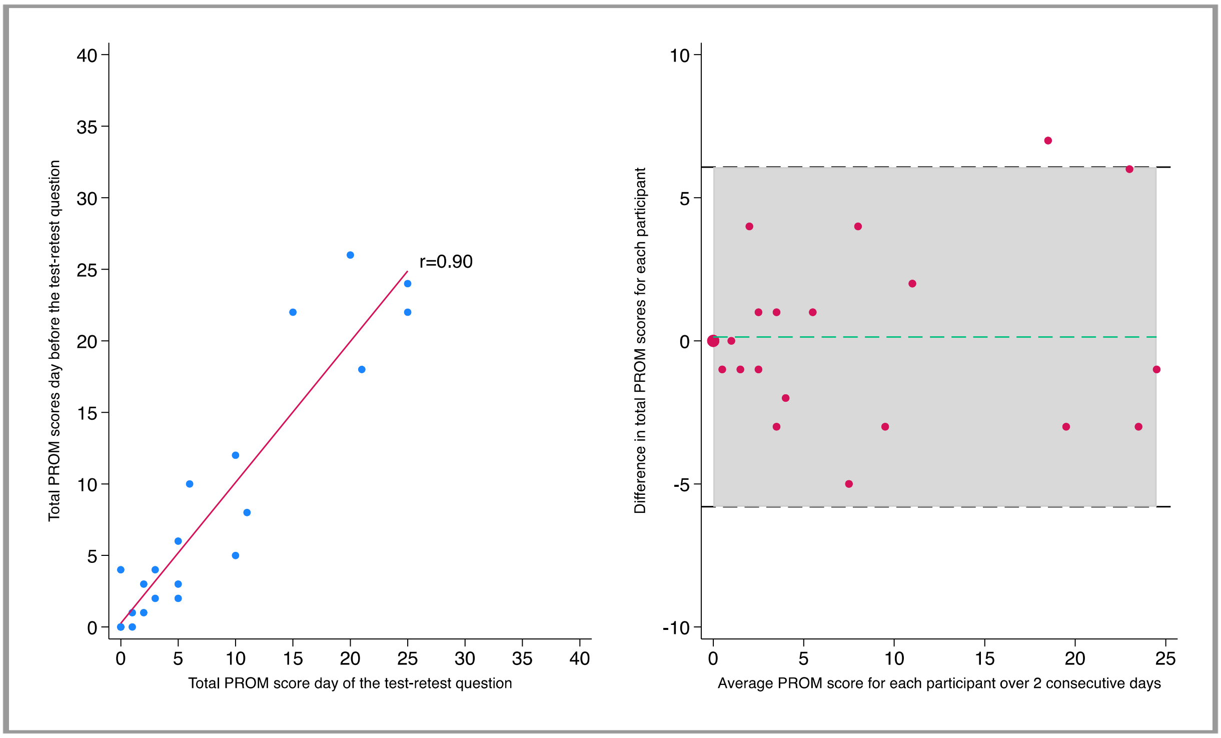

Supplementary material ten. Assessment of test-retest reliability in those reporting stable symptoms. Left: Spearman's correlation coefficient demonstrated a positive correlation of total scores on two consecutive days. Right: Bland-Altman plot of the mean difference in total scores for participants across two consecutive days. Test-retest question was "How much have your tummy symptoms bothered you today compared to yesterday?".

Supplementary material 11

Item	Percent agreement	p-value	95% CI for percent agreement	Gwet's AC	p-value	95% CI for Gwet's AC
Tummy discomfort	0.68	<0.001	0.47, 0.89	0.62	<0.001	0.36, 0.88
Unpredictability	0.59	<0.001	0.37, 0.81	0.51	0.001	0.23, 0.79
Nausea	0.91	<0.001	0.78, 1.0	0.89	<0.001	0.72, 1.0
Sleep	0.91	<0.001	0.78, 1.0	0.89	<0.001	0.72, 1.0
Embarrassed	0.64	<0.001	0.42, 0.85	0.56	<0.001	0.29, 0.84
Impact	0.77	0.001	0.58, 0.96	0.74	<0.001	0.51, 0.97
Bloating	0.68	<0.001	0.47, 0.89	0.62	<0.001	0.35, 0.88
Concentration	0.86	<0.001	0.71, 1.0	0.85	<0.001	0.67, 1.0
Stool consistency	0.55	<0.001	0.32, 0.77	0.44	0.004	0.16, 0.73
Eating	0.55	<0.001	0.32, 0.77	0.43	0.01	0.12, 0.74

Supplementary material 11. Percentage agreement and Gwet's AC for assessment of test-retest reliability of individual question scores across two consecutive days.
