# Supplementary material for: CF Tummy Tracker: A Cystic Fibrosis–Specific Patient-Reported Outcome Measure for Daily Gastrointestinal Symptom Burden
Source: Mayo Clin Proc Digit Health. 2025 Mar 3;3(2):100203. doi: 10.1016/j.mcpdig.2025.100203 (PMC12191005; doi:10.1016/j.mcpdig.2025.100203)
Supplement: Supplementary Data [file mmc2.docx]

**Manuscript title:** CF Tummy Tracker: A cystic fibrosis-specific patient reported outcome measure for daily gastrointestinal symptom burden

**Article type:** Original research

**Corresponding author:** Dr Rebecca Calthorpe

**Role of the funding source:** This study was funded by the National Institute for Health and Care Research Programme Development Grant (NIHR202952). The funder had no involvement in the study design; collection, analysis or interpretation of the data; manuscript preparation or decision to submit the manuscript for publication.

**Authors contributions**

Rebecca Calthorpe: Conceptualisation, data curation, formal analysis, investigation, project administration, visualization, writing original draft,

Hisham Saumtally: data curation,investigation, project administration, writing - review and editing

Laura Howells: Conceptualisation, data curation, supervision, writing - review and editing

Natalie Goodchild: Conceptualisation, writing - review and editing, patient representative

Bethinn Evans: Conceptualisation, writing - review and editing, patient representative

Zoe Elliott: Conceptualisation, writing - review and editing, patient representative

Siobhán Carr: Conceptualisation, fund acquisition, resources, writing - review and editing

Caroline Elston: Resources, writing - review and editing

Alexander Horsley: Conceptualisation, fund acquisition, resources, writing - review and editing

Daniel Peckham: Conceptualisation, fund acquisition, resources, writing - review and editing

Helen Barr: Conceptualisation, Resources, writing - review and editing

Bu Hayee: Conceptualisation, Resources, writing - review and editing

Giles Major: Conceptualisation, fund acquisition,

Iain Stewart: Conceptualisation, fund acquisition, formal analysis, methodology, supervision, writing - review and editing

Kim Thomas: Conceptualisation, fund acquisition, formal analysis, methodology, supervision, writing - review and editing

Alan Smyth: Conceptualisation, fund acquisition, formal anal sis, methodology, supervision, writing - review and editing
